# Efficacy and safety of flow diverters in basilar artery aneurysms: a single-center retrospective cohort study

**DOI:** 10.3389/fneur.2025.1668097

**Published:** 2025-10-22

**Authors:** Ziyuan Huang, Chuan Chen, Baoyu Zhang, Yuanjun Hu, Hui Wang, Cong Ling

**Affiliations:** Department of Neurosurgery, The Third Affiliated Hospital, Sun Yat-sen University, Guangzhou, China

**Keywords:** flow diverter, basilar artery aneurysm, endovascular treatment, safety, efficacy

## Abstract

**Objective:**

To evaluate the efficacy and safety of flow diverter (FD) devices in the treatment of basilar artery aneurysms, and to assess their clinical outcomes and associated complications.

**Methods:**

A retrospective analysis was conducted on 30 patients with basilar artery aneurysms treated with FD devices at our institution between 2020 and 2024. Patient demographics, aneurysm characteristics, intraoperative and postoperative imaging, and clinical follow-up data were collected. Statistical analysis was performed to assess treatment efficacy and complication rates.

**Results:**

Among the 30 patients, 20 were male (66.7%) and 10 were female (33.3%), with a median age group of 65–69 years. The majority of aneurysms were located in the basilar artery trunk (70%), while 30% were basilar apex aneurysms. All procedures were technically successful (success rate: 100%). The mean aneurysm diameter was 10.6 ± 4.9 mm. The mean follow-up period was 12.9 months. Imaging follow-up demonstrated a complete or near-complete aneurysm occlusion rate of 86.7%; occlusion rates for the Tubridge and Pipeline Flex devices were 83.3 and 88.9%, respectively, with no statistically significant difference between the two devices (*p* > 0.05). Treatment-related complications occurred in 4 cases (13.3%), all presenting as transient ischemic symptoms, with no cases of permanent severe neurological deficits. 93.3% of patients (28/30) achieved an excellent functional outcome (mRS score of 0–1), and all patients (100%) had a functional outcome of mRS 0–2.

**Conclusion:**

In this retrospective cohort, FD treatment for basilar artery aneurysms was associated with a high rate of complete or near-complete occlusion (86.7%) and a favorable safety profile, as evidenced by the low rate of complications (13.3%, all transient). The clinical outcomes were excellent, with 93.3% of patients achieving an mRS of 0–1. The Tubridge and Pipeline devices demonstrated comparable efficacy and safety outcomes in this study, although the small sample size and non-randomized design preclude definitive conclusions regarding superiority or equivalence. Our findings indicate that rigorous preoperative antiplatelet management and meticulous operative technique are critical for these results. This study supports the consideration of FDs for BAAs in carefully selected patients, but further large-scale, prospective studies are warranted to confirm long-term durability and optimize patient selection.

## 1 Introduction

Basilar artery aneurysms (BAAs), a distinct and complex subtype of intracranial aneurysms, account for approximately 7%–8% of all intracranial aneurysms and nearly 50% of aneurysms within the posterior circulation (1, 2). Among these, basilar trunk aneurysms represent 0.95%–2.1% of all intracranial aneurysms (3, 4). Typically located in a deep-seated area anterior to the brainstem and adjacent to critical perforating arteries and major branches—such as the superior cerebellar artery (SCA), anterior inferior cerebellar artery (AICA), and posterior cerebral artery (PCA)—the complex anatomy of BAAs presents considerable therapeutic challenges. Given their high morbidity and mortality rates, prompt and effective management is crucial for improving patient outcomes and minimizing the risks of disability and death (5, 6).

Traditional microsurgical approaches are limited by the deep and compact location of the basilar artery and its proximity to the brainstem, resulting in substantial exposure risks and a high likelihood of neural injury, making them less than ideal. Endovascular coil embolization, which has been considered the “gold standard” for posterior circulation aneurysms (7), is associated with a higher risk of recurrence and complications, especially in large aneurysms (8, 9). With advancements in endovascular technology, flow diverters (FDs) with fine mesh designs have emerged as a minimally invasive alternative, achieving aneurysm occlusion by modulating local hemodynamics, promoting endothelialization of the aneurysm neck, and inducing thrombosis, as well as straightening the parent artery (10, 11). The Pipeline Embolization Device (PED; Medtronic, USA), the first FD approved for clinical use, currently has the most robust clinical and laboratory evidence (12). Composed of cobalt-chromium (75%) and platinum (25%), the PED offers improved radiopacity due to higher stiffness, though this rigidity can hinder complete deployment in tortuous vascular segments (13). The Tubridge flow diverter (TFD; MicroPort, China) is a relatively newer device comprised of a self-expanding, braided nitinol stent with flared ends, exhibiting favorable outcomes in the treatment of unruptured intracranial aneurysms (UIAs) and offering advantages of superelasticity and shape memory (14, 15). Compared to PED, the nitinol design of TFD provides greater flexibility, allowing better trackability, though the lower radial force may predispose the device to migration during microcatheter or microwire manipulation (16, 17). The reported aneurysm occlusion rate for FDs is about 90.9%, superior to traditional coil embolization techniques (18). However, the unique anatomical and physiological characteristics of BAAs raise specific challenges for FD application. Coverage of the basilar artery and its critical perforators can compromise blood supply, increasing the risk of ischemic stroke and new neurological deficits. Additionally, the efficacy of FDs in treating large or fusiform aneurysms remains controversial, with some studies reporting lower complete occlusion rates compared to smaller aneurysms. Factors such as patient-specific anatomy, aneurysm morphology, antiplatelet regimens, and intraprocedural technical aspects significantly affect both safety and outcomes.

Given these considerations, we conducted a comprehensive retrospective analysis of consecutive patients with BAAs treated with FD devices at our center. This study aims to systematically evaluate the procedural success, aneurysm occlusion rates, complications, and functional outcomes associated with FD treatment, thereby providing robust evidence to guide individualized management of basilar artery aneurysms and to inform future strategies for device and technique optimization in clinical practice.

## 2 Materials and methods

### 2.1 Study design

**Figure 1:**
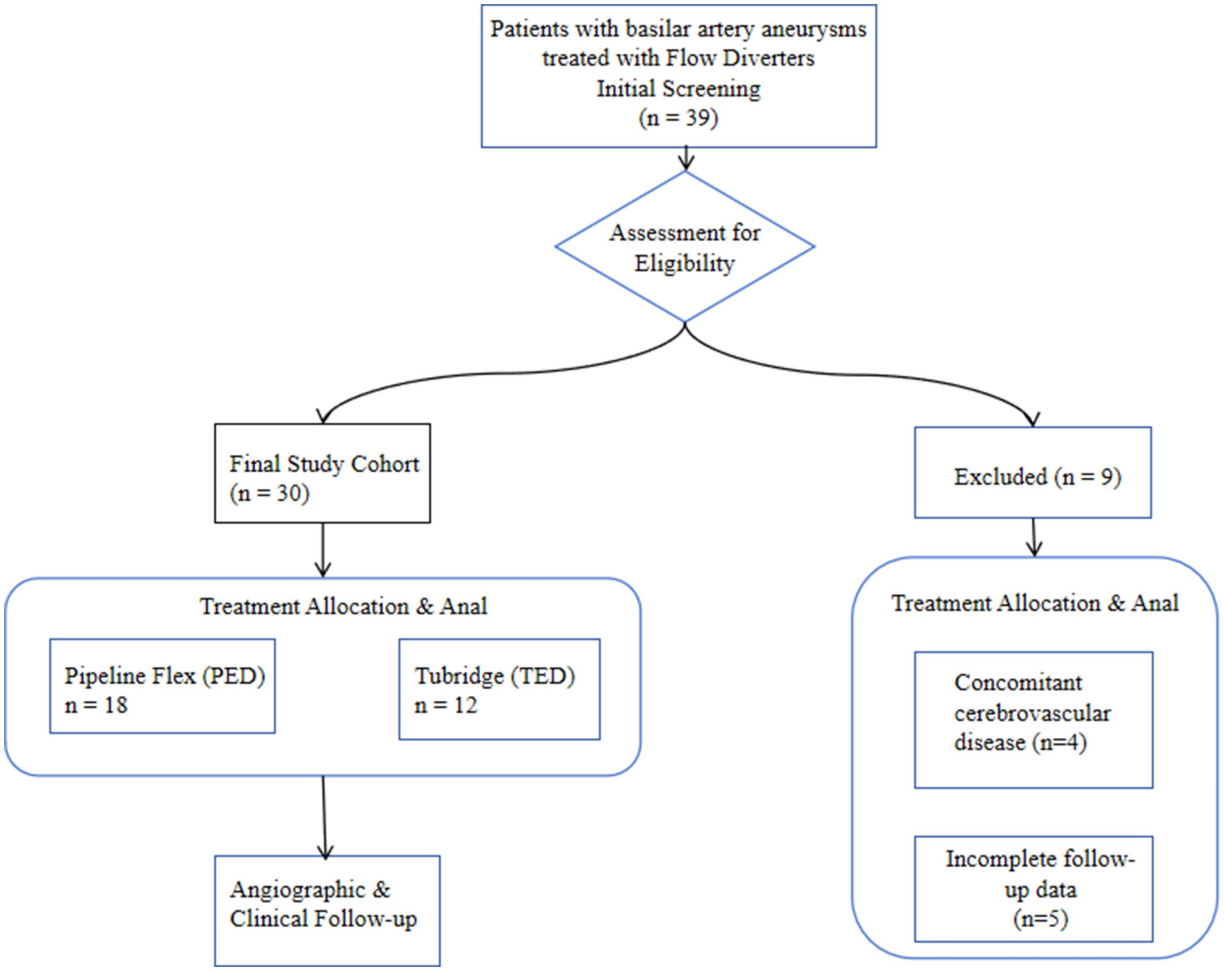
Flowchart of patient selection and study cohort formation.

We performed a single-center, retrospective cohort study of 30 patients with basilar artery aneurysms treated with flow diverters (FDs) between January 2020 and September 2024. The patient screening, eligibility assessment, and inclusion process are detailed in the study flowchart (Figure 1). The institutional review board approved the protocol and waived informed consent due to the study’s retrospective nature and anonymized data collection. Inclusion criteria were: (1) unruptured intracranial aneurysm confirmed by DSA or CTA; (2) treatment with a Pipeline Embolization Device (PED) or Tubridge (TFD); and (3) ≥8 months of post-procedural imaging follow-up (DSA, CTA, or MRI). Exclusion criteria included concomitant arteriovenous malformations, moyamoya disease, or other cerebrovascular disorders, and incomplete follow-up data. Demographic, clinical, aneurysm morphology, procedural, angiographic and clinical outcome data were extracted from electronic medical records.

### 2.2 Surgical procedure

All patients received a standardized preprocedural dual antiplatelet therapy regimen comprising aspirin (100 mg daily) and clopidogrel (75 mg daily) for a minimum of 5 days prior to the intervention. In accordance with our institutional protocol for flow diverter procedures, all patients underwent comprehensive preoperative platelet function assessment through thromboelastography and the Verify Now P2Y12 assay to ensure adequate and individualized platelet inhibition. Clopidogrel hyporesponse was defined as < 30% platelet inhibition on the Verify Now P2Y12 assay. Patients identified as clopidogrel hyporesponders, defined by ADP-induced platelet inhibition below 30% on Verify Now testing, were transitioned to ticagrelor therapy with a loading dose of 180 mg followed by a maintenance dose of 90 mg twice daily. This individualized approach ensured optimal platelet inhibition while minimizing thromboembolic and hemorrhagic risks. Postoperative antiplatelet therapy was maintained for at least 6 months, with ongoing monitoring in selected cases. Under general anesthesia and via a right femoral approach, a triaxial system (6 Fr Neuron MAX guide catheter, 5 Fr Sofia EX intermediate catheter, Marksman microcatheter) was used to deploy the FD. Intravenous heparin (70–80 U/kg) was administered to maintain activated clotting time at 2–2.5 × baseline. Device choice and sizing were based on aneurysm geometry and operator experience; aneurysms > 15 mm or with persistent inflow jet underwent adjunctive coiling (Micro Vention). Following deployment, DynaCT (Artis Q, Siemens) confirmed wall apposition and excluded acute complications. Post-procedure, dual antiplatelet therapy was continued for 6 months.

### 2.3 Follow-up

Neurological status was assessed at discharge using the modified Rankin Scale (mRS). Imaging follow-up consisted of high-resolution 3 T TOF-MRA and DSA. Aneurysm occlusion was graded by the O’Kelly-Marotta (OKM) scale (D = complete occlusion; C = neck remnant; B = subtotal filling; A = no occlusion). Branch vessel patency was evaluated on CTA or quantitative vessel analysis (Philips IntelliSpace Portal), with ≥ 50% stenosis defined as significant.

### 2.4 Statistical analysis

Categorical variables are presented as counts and percentages. Continuous variables were tested for normality using the Shapiro–Wilk test and are presented as mean ± standard deviation or median with interquartile range, as appropriate. Between-group comparisons were performed using Pearson’s χ^2^ test or Fisher’s exact test for categorical variables. For continuous variables, Student’s *t*-test was used for normally distributed data, and the Mann–Whitney U test was used for non-normally distributed data. Univariate and multivariate logistic regression analyses were conducted to identify factors associated with complications, occlusion outcomes, and poor clinical outcome (defined as mRS ≥ 3). For the multivariate model, variables with a *p*-value < 0.1 in univariate analysis and those deemed clinically relevant were included. A two-sided *p* < 0.05 was considered statistically significant. All statistical analyses were performed using SPSS version 26.0 (IBM Corp., Armonk, NY, USA).

## 3 Results

Between January 2020 and September 2024, a total of 39 patients with basilar artery aneurysms were treated with flow-diverting stents at our neurointerventional center. Based on the predefined criteria, 9 patients were excluded, resulting in a final study cohort of 30 patients eligible for analysis. The detailed process of patient selection is illustrated in Figure 1. Thirty patients (median age group 65–69 years; 20 men, 10 women) harboring basilar artery aneurysms were included (Table 1). The mean aneurysm diameter was 10.6 ± 4.9 mm. Twenty-two patients (73.3%) were over 60 years. Presentation was dizziness in 7 (23.3%), headache in 2 (6.7%), brainstem compression in 3 (10%), and incidental in 6 (20%). Three patients (10%) had recurrent aneurysms after prior coiling. At presentation, mRS was 0 in 22 (73.3%), 1 in 5 (16.7%), and ≥ 2 in 3 (10%). Aneurysm location was the basilar trunk in 21 (70%) and the basilar tip in 9 (30%). Patient demographics, aneurysm characteristics, and treatment details are summarized in Table 2, with statistical comparisons provided.

**Table 1:** Patient demographics, aneurysm characteristics, treatment details, and individual OKM occlusion grades.

Pt #	Age, y (sex)	Presentation	mRS (Pre)	Previous treatment	Aneurysm location	Aneurysm diameter (mm)	Number and type of FDs used	Additional devices applied	Jailed major branches	Complications	Angiographic FU time (months)	Angiographic FU modality	Occlusion grade	OKM grade (A/B/C/D)	Jailed branches status	mRS at FU
1	50–54 (M)	Dizziness	0	–	Trunk	5.3 × 3.7	1 PED	–	1 AICA	–	12	DSA	CO	D	Patent	0
2	65–69 (M)	–	0	–	Trunk	11 × 10	1 PED	–	1 AICA	–	15	DSA	CO	D	Patent	0
3	65–69 (F)	Dizziness	0	–	Trunk	15 × 14	1 TFD	–	1AICA	–	10	DSA	CO	D	Patent	0
4	65–69 (M)	–	0	–	Trunk	11 × 10	1 TFD	–	1AICA	–	9	DSA	CO	D	Patent	0
5	75–79(M)	–	0	–	Trunk	3.5 × 3.0	1 PED	–	1 AICA	–	12	DSA	CO	D	Patent	0
6	70–74 (M)	–	0	–	Trunk	9 × 23	1 TFD	–	1 AICA	–	8	DSA	CO	D	Patent	0
7	65–69 (M)	Brainstem compression	1	–	Trunk	12 × 8	1 PED	–	1 SCA, 1 PCA	–	13	DSA	CO	D	Patent	0
8	65–69 (M)	Dizziness	0	–	Tip	13 × 22	1 PED	–	1 PCA	–	9	DSA	NC	C	Patent	0
9	75–79 (M)	Headache	0	–	Tip	8.3 × 8.5	1 PED	–	1 PCA, 1 SCA	–	13	DSA	CO	D	Patent	0
10	55–59 (F)	Recurrence after previous treatment	1	Coiling	Trunk	7.2 × 6.8	1 PED	Coil	1 AICA	TIA	14	DSA	NC	C	Occluded	0
11	60–64 (M)	Dizziness	2	–	Trunk	18 × 15	2 TFD	Coil	1AICA	–	16	DSA	PO	B	Patent	1
12	45–49 (F)	Dizziness	0	–	Tip	5.5 × 4.2	1 PED	–	1 PCA	–	11	CTA	NC	C	Patent	0
13	70–74 (M)	Syncope	0	–	Trunk	14 × 11	1 PED	–	1 SCA, 1 PCA	TIA	18	DSA	NC	C	Occluded	2
14	55–59 (F)	–	0	–	Tip	4.8 × 3.9	1 TFD	–	1 PCA	–	10	DSA	CO	D	Patent	0
15	65–69 (M)	–	0	–	Trunk	10 × 8	1 PED	–	1AICA	–	12	DSA	CO	D	Patent	0
16	50–54 (F)	–	0	–	Tip	6.7 × 5.5	1 PED	–	1 PCA	–	14	DSA	NC	C	Patent	0
17	70–74 (M)	Cranial nerve palsy	1	–	Trunk	20 × 18	2 PED	–	1 AICA	–	17	DSA	PO	B	Patent	0
18	60–64 (F)	Recurrence after previous treatment	1	Coiling	Tip	9.1 × 7.3	1 TFD	–	1 PCA	–	13	DSA	CO	D	Patent	0
19	60–64 (M)	–	0	–	Trunk	8.2 × 6.5	1 PED	–	1 AICA	–	11	DSA	NC	C	Patent	0
20	55–59 (F)	Dizziness	1	–	Tip	12 × 10	1 TFD	Coil	1 PCA	–	15	DSA	NC	C	Patent	1
21	70–74 (M)	–	0	–	Trunk	7.8 × 6.2	1 PED	–	1AICA	–	10	DSA	CO	D	Patent	0
22	55–59 (F)	–	1	–	Tip	13 × 11	1 TFD	–	1 PCA	–	19	MRI	NC	C	Patent	1
23	60–64 (M)	Tinnitus	0	–	Trunk	6.5 × 5.0	1 PED	–	1AICA	–	12	DSA	CO	D	occluded	0
24	60–64 (F)	Recurrence after previous treatment	3	Embolization	Tip	22 × 19	2 TFD	–	2 PCA	TIA	20	DSA	PO	B	Patent	2
25	55–59 (M)	Brainstem compression	0	–	Trunk	9.5 × 7.8	1 PED	–	1 AICA	–	14	DSA	CO	D	Patent	0
26	60–64 (M)	Headache	0	–	Trunk	12.5 × 10.2	1 PED	–	1 AICA	–	10	DSA	PO	B	Patent	00
27	70–74 (F)	70–74 (F)	0	–	Tip	7.8 × 6.5	1 TFD	–	1 PCA	–	9	DSA	NC	C	Patent	0
28	65–69 (M)	Dizziness	1		Trunk	9.2 × 8.1	1 PED	–	1 AICA	TIA	10	DSA	CO	D	Patent	1
29	55–59 (F)	–	0		Tip	6.5 × 5.8	1 TFD	–	1 PCA	––	12	DSA	CO	D	Patent	0
30	75–79 (M)	Brainstem compression	2	–	Trunk	14.7 × 11.7	2 TFD	Coil	1 AICA, 1 PCA	–	18	DSA	NC	C	Patent	1

OKM Occlusion Grade: D = Complete occlusion; C = Near-complete occlusion (neck remnant); B = Partial occlusion (subtotal filling); A = No occlusion. PED, pipeline embolization device; TFD, tubridge flow diverter; mRS, modified rankin scale; FU, follow-up; TIA, transient ischemic attack; AICA, anterior inferior cerebellar artery; PCA, posterior cerebral artery; SCA, superior cerebellar artery; DSA, digital subtraction angiography; CTA, computed tomography angiography; MRI, magnetic resonance imaging.

**Table 2:** Comparative analysis of baseline characteristics and outcomes between PED and TFD treatment groups.

Parameter	Overall (*n* = 30)	PED group (*n* = 18)	TFD group (*n* = 12)	*p*-value
Demographics				
Age, years, median (IQR)	65 (60–69)	65 (60–69)	66.5 (60.75–71)	0.536^a^
Male sex, *n* (%)	20 (66.7)	13 (72.2)	7 (58.3)	0.462^b^
Presentation, *n* (%)				
Incidental	6 (20.0)	4 (22.2)	2 (16.7)	1.000^b^
Dizziness	7 (23.3)	4 (22.2)	3 (25.0)	1.000^b^
Clinical status				
Preoperative mRS ≥ 2, *n* (%)	3 (10.0)	1 (5.6)	2 (16.7)	0.547^b^
Recurrent sneurysm, *n* (%)	4 (13.3)	1 (5.6)	3 (25.0)	0.273^b^
Aneurysm characteristics				
Location: trunk, *n* (%)	21 (70.0)	13 (72.2)	8 (66.7)	1.000^b^
Location: tip, *n* (%)	9 (30.0)	5 (27.8)	4 (33.3)	1.000^b^
Size, mm, mean ± SD	10.6 ± 4.9	9.7 ± 3.8	12.0 ± 6.1	0.198^c^
Treatment details				
Adjunctive coiling, *n* (%)	4 (13.3)	1 (5.6)	3 (25.0)	0.273^b^
Number of jailed branches, median (IQR)	1 (1–2)	1 (1–1)	1 (1–1.25)	0.699^a^
Complications				
Transient ischemic symptoms (TIA), n (%)	4 (13.3)	2 (11.1)	2 (16.7)	1.000^b^
Follow-up				
Angiographic FU time, months, mean ± SD	12.9 ± 3.5	12.6 ± 2.7	13.4 ± 4.5	0.539^c^
Occlusion outcome, *n* (%)				
Complete/near-complete (CO/NC)	26 (86.7)	16 (88.9)	10 (83.3)	1.000^b^
Partial (PO)	4 (13.3)	2 (11.1)	2 (16.7)	1.000^b^
Jailed branch occlusion at FU, *n* (%)	3 (10.0)	2 (11.1)	1 (8.3)	1.000^b^
Clinical outcome at FU, *n* (%)				
mRS 0–1	28 (93.3)	17 (94.4)	11 (91.7)	1.000^b^
mRS 2	2 (6.7)	1 (5.6)	1 (8.3)	1.000^b^

Data are presented as *n* (%), mean ± standard deviation, or median (interquartile range – IQR). PED, pipeline embolization device; TFD, tubridge flow diverter; mRS, modified rankin scale; FU, follow-up; TIA, transient ischemic attack; CO, complete occlusion; NC, near-complete occlusion; PO, partial occlusion.

a Mann–Whitney *U* test.

b Fisher’s Exact Test.

c Independent samples *t*-test.

**Figure 2:**
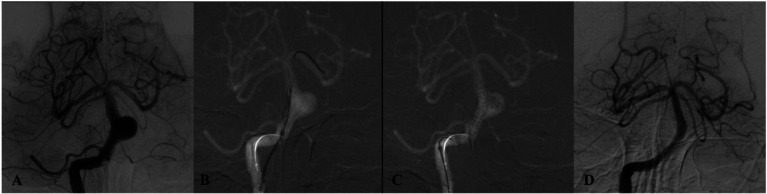
DSA of a patient from the 50–54 age group (Case 1) presenting with dizziness. **(A)** Preprocedural DSA demonstrates a saccular aneurysm of the basilar trunk measuring 5.3 × 3.7 mm. **(B,C)** Lateral and anteroposterior views immediately after deployment of a single PED across the aneurysm neck. **(D)** 12-month follow-up DSA confirms complete aneurysm occlusion.

**Figure 3:**
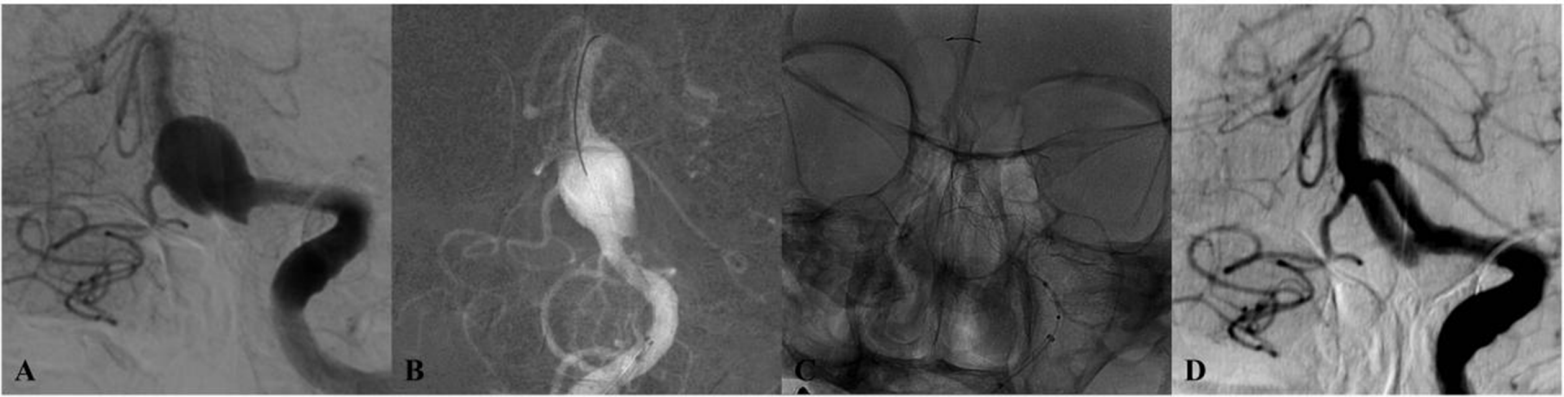
DSA of a patient from the 65–69 age group (Case 3) with dizziness. **(A)** Initial angiogram shows a giant basilar trunk aneurysm measuring 15 × 14 mm. **(B,C)** Post-treatment lateral and anteroposterior projections after implantation of a single Tubridge flow diverter. **(D)** 10-month follow-up DSA reveals durable, complete occlusion.

**Figure 4:**
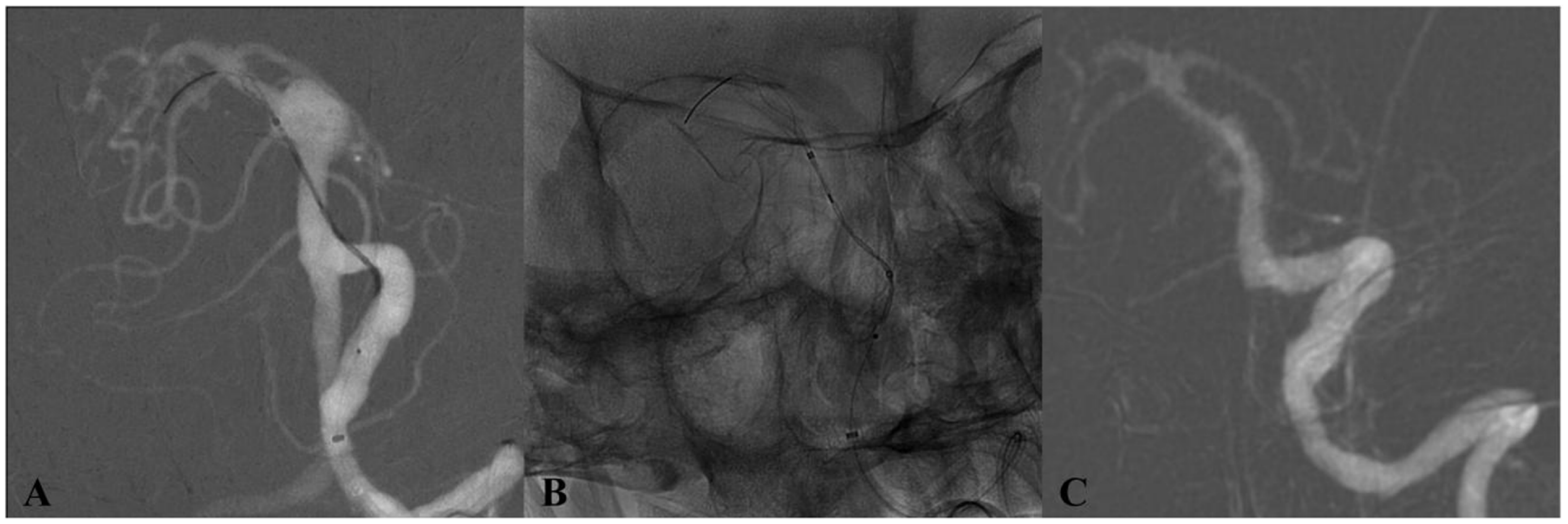
DSA of a patient from the 65–69 age group (Case 4) with an incidentally discovered basilar trunk aneurysm. **(A)** Preoperative angiography depicts an 11 × 10 mm aneurysm at the basilar trunk. **(B)** Immediate postdeployment DSA following placement of a single Tubridge flow diverter. **(C)** 17-month follow-up DSA demonstrates full aneurysm occlusion.

Technical success was 100%; all flow diverters (FDs) fully covered the aneurysm neck. Eighteen patients (60.0%) received a Pipeline Flex (PED), and 12 patients (40.0%) received a Tubridge (TFD); three cases required two TFDs, and one required two PEDs. Adjunctive coiling was performed in four cases. Deployment was uneventful in all procedures. In total, 36 major branches were jailed: 17 AICAs, 3 SCAs, and 16 PCAs. Four patients (13.3%) experienced transient ischemic symptoms (three with unilateral limb numbness; one with numbness and diplopia), all of which resolved completely with vasospasm therapy before discharge. No acute branch occlusions were observed. Radiologic follow-up was available in 28 patients with DSA and in two patients with MRA or CTA. The mean duration of angiographic follow-up was 12.9 months (range, 8–20 months). Representative cases are shown in Figures 2–4. Factors associated with complete occlusion, complications, and clinical outcomes were systematically analyzed (Table 3). In univariate analysis, larger aneurysm size was significantly associated with lower odds of complete occlusion (OKM grade C/D; OR 0.47, 95% CI 0.14–0.77, *p* = 0.042). Multivariate Firth regression, which included aneurysm size, age, and adjunctive coiling, confirmed aneurysm size as an independent predictor of complete occlusion (OR 0.62, 95% CI 0.29–0.85, *p* < 0.001). For TIA complications, no significant associations were identified with aneurysm size (*p* = 0.2), age (*p* > 0.9), or other factors. Regarding favorable clinical outcome (mRS 0–1), larger aneurysm size showed a trend toward significance (OR 0.67, 95% CI 0.38–0.94, *p* = 0.055), while other factors including age, sex, aneurysm location, device type, and adjunctive coiling demonstrated no significant associations. At last follow-up, complete or near-complete aneurysm occlusion (CO or NC) was achieved in 16 of 18 PED-treated patients (88.9%) and in 10 of 12 TFD-treated patients (83.3%), resulting in an overall rate of 86.7% (26/30). Partial occlusion (PO) was seen in two PED cases (11.1%) and two TFD cases (16.7%). The difference in complete/near-complete occlusion rates between the two devices was compared using Fisher’s exact test due to the small sample size. The analysis revealed no statistically significant difference (*p* = 1.000). It is important to note that this high *p*-value likely reflects the limited statistical power of the study to detect differences given the small cohort sizes, particularly for the TFD group (*n* = 12), rather than definitive evidence of equivalence. At a mean clinical follow-up of 12.9 months, 22 patients (73.3%) had an mRS of 0, 6 patients (20.0%) had an mRS of 1, and 2 patients (6.7%) had an mRS of 2 at last assessment. Angiographic follow-up revealed occlusion of three jailed branches (one AICA and two PCAs, 10.0%) in three patients (Cases 10, 13, and 23).

**Table 3:** Multivariable analysis of factors associated with treatment outcomes.

Predictor	Complete occlusion (OKM C/D)		TIA Complications		Favorable clinical outcome (mRS 0–1)	
	OR (95% CI)	*p*-value	OR (95% CI)	*p*-value	OR (95% CI)	*p*-value
Aneurysm size, per mm						
Univariate	0.47 (0.14–0.77)	**0.042**	1.15 (0.92–1.48)	0.20	0.67 (0.38–0.94)	0.055
Multivariate^a^	0.62 (0.29–0.85)	**< 0.001**	–	–	–	–
Age, per year	0.99 (0.86–1.14)	0.90	1.01 (0.88–1.16)	0.90	0.95 (0.75–1.15)	0.60
Male sex	0.53 (0.02–4.85)	0.60	0.53 (0.06–5.02)	0.60	1.80 (0.07–48.9)	0.70
Basilar trunk location	0.53 (0.02–4.85)	0.60	1.87 (0.21–40.9)	0.60	1.80 (0.07–48.9)	0.70
TFD device	0.63 (0.07–5.89)	0.70	0.45 (0.02–4.11)	0.50	0.65 (0.02–17.5)	0.80
Adjunctive coiling	0.39 (0.03–9.30)	0.50	2.56 (0.11–29.7)	0.50	^b^	> 0.99
Preoperative mRS ≥ 2	0.39 (0.03–9.30)	0.50	2.56 (0.11–29.7)	0.50	^b^	> 0.99

a Multivariate analysis using Firth’s penalized logistic regression (variables: aneurysm size, age, adjunctive coiling).

b Complete separation prevented reliable estimation due to all patients with adjunctive coiling/preoperative mRS ≥ 2 achieving favorable outcomes.

OKM C/D, complete or near-complete occlusion; TIA, transient ischemic attack; mRS, modified Rankin Scale. Bold values indicate statistical significance (*p* < 0.05).

Univariate analysis identified aneurysm size as significantly associated with complete occlusion (OKM Grade C/D) (OR = 0.47, 95% CI: 0.14–0.77, *p* = 0.042). Age, sex, aneurysm location, device type (PED vs. TFD), and the use of adjunctive coiling did not demonstrate significant associations in the univariate model (all *p* > 0.05). Multivariate analysis using Firth logistic regression confirmed that smaller aneurysm size was an independent predictor of complete occlusion (adjusted OR = 0.62, 95% CI: 0.29–0.85, *p* < 0.001). After adjustment, neither age (adjusted OR = 1.10, 95% CI: 0.88–1.66, *p* = 0.4) nor adjunctive coiling (adjusted OR = 0.85, 95% CI: 0.04–90.4, *p* > 0.9) showed significant association with the occlusion outcome. Four patients (13.3%) experienced transient ischemic attacks (TIA). Univariate analysis revealed no significant associations between TIA occurrence and patient age (OR = 1.01, *p* > 0.9), sex (OR = 0.53, *p* = 0.6), aneurysm size (OR = 1.15, *p* = 0.2), location (OR = 1.87, *p* = 0.6), device type (OR = 0.45, *p* = 0.5), or adjunctive coiling (OR = 2.56, *p* = 0.5). Twenty-eight patients (93.3%) achieved favorable clinical outcomes (mRS 0–1) at follow-up. Univariate analysis showed no significant associations between clinical outcome and age (OR = 0.95, *p* = 0.6), sex (OR = 1.80, *p* = 0.7), aneurysm size (OR = 0.67, *p* = 0.055), location (OR = 1.80, *p* = 0.7), device type (OR = 0.65, *p* = 0.8), adjunctive coiling, or TIA complications. Aneurysm size showed a trend toward significance but did not reach the statistical threshold (*p* = 0.055).

## 4 Discussion

BAAs present a formidable challenge in neurointerventional surgery, attributable to their deep-seated location adjacent to the brainstem, their proximity to critical perforating arteries, and complex regional hemodynamics. While microsurgical clipping has been largely superseded for these lesions due to associated high morbidity and mortality, the advent of FDs has markedly transformed the therapeutic landscape. In our retrospective cohort of 30 consecutive BAA patients treated with FDs, we observed a 100% technical success rate, an overall complete or near-complete occlusion rate of 86.7% at a mean follow-up of 12.9 months, and a symptomatic complication rate of 13.3% comprising transient ischemic events. These outcomes underscore the considerable promise of FD technology for managing these complex aneurysms.

He complete or near-complete occlusion rate of 86.7% achieved in our FD-treated cohort compares favorably with historical outcomes reported for conventional endovascular techniques. As summarized in Table 4, large meta-analyses of conventional coil embolization for posterior circulation aneurysms report complete occlusion rates ranging from 70.0% to 72.9% (19–21). Stent-assisted coiling (SAC) exhibits variable efficacy, with reported rates between 59.2 and 81% (22–24). Similarly, microsurgical clipping, though highly effective in selected cases, is associated with significant risks due to the complex anatomy of the basilar artery, with reported complete occlusion rates ranging from 80% to 90% but at the cost of higher morbidity and mortality rates (21, 25). In contrast, contemporary FD series, including the present study, consistently report mid-term complete or near-complete occlusion rates between 77% and 88.3% (22, 26–30), positioning FDs as a highly effective modality. This consistent performance highlights the fundamental mechanistic advantage of FDs: parent vessel reconstruction and intra-aneurysmal flow disruption promote progressive thrombosis and endothelialization across the neck, a mechanism distinct from simply filling the aneurysm sac with coils.

**Table 4:** Comprehensive literature overview of occlusion outcomes following various treatment modalities for basilar artery aneurysms.

Study (year)	Treatment method	Aneurysm characteristics	Sample size	Follow-up (months)	Complete occlusion rate (%)	Evidence level
Lozier et al. (2002) (19)	Coil embolization	Posterior circulation	495	N/R	72.9	I
Henkes et al. (2005) (20)	Coil embolization	Basilar apex aneurysms	316	19.0	70.0	I
Spiessberger et al. (38)	Microsurgical clipping	Basilar apex aneurysms	1,764	N/R	93.0	I
Luo et al. (2024) (22)	Stent-assisted coiling (SAC)	Basilar artery aneurysms	88	7.6	59.2%	III
Zhang et al. (2013) (23)	Stent-assisted coiling (SAC)	Wide-necked basilar artery bifurcation aneurysms	23	13.5	62.5	III
Nejadhamzeeigilani et al. (2023) (24)	Stent-assisted coiling (SAC)	Wide-necked basilar tip aneurysms	19	32	81	III
Srinivasan et al. (2024) (28)	Flow diverter (FD)	Basilar quadrifurcation aneurysms	34	6.6	88	III
Luo et al. (2024) (22)	FD (pipeline)	Basilar artery aneurysms	51	7.6	86.7%	III
Wang et al. (2024) (29)	FD	Basilar artery aneurysms	16	7.7	86.7	III
Qi et al. (2023) (30)	FD	Basilar artery aneurysms	33	29.5	63	III
Present study (2025)	Flow diverter (FD)	Basilar artery aneurysms	30	13.1	88.3 (Complete/near-complete)	III

Evidence Level: I = Meta-analysis; II = Prospective cohort; III = Retrospective cohort.

N/R, Not reported.

Follow-up duration presented as mean or median values as reported in original studies.

Aneurysm size is a well-established factor influencing occlusion outcomes after FD treatment. A recent meta-analysis of the Surpass Evolve flow diverter demonstrated an inverse relationship between aneurysm diameter and complete occlusion rate (31), consistent with the hemodynamic principle that flow disruption is more effective in smaller aneurysms with less voluminous inflow zones. In our cohort, the mean aneurysm diameter was 10.6 mm, and the high occlusion rate likely reflects appropriate patient selection based on morphological characteristics. Our multivariate analysis further identified larger aneurysm size as an independent predictor for lower odds of complete occlusion (adjusted OR = 0.62, *p* < 0.001), aligning with established literature. This reinforces the importance of meticulous case selection to optimize outcomes.

In our series, both the Pipeline Flex and Tubridge devices achieved high and comparable mid-term complete or near-complete occlusion rates (88.9% vs. 83.3%, *p* = 1.0). However, this lack of statistical significance must be interpreted with caution due to the limited sample size, particularly the smaller Tubridge subgroup (*n* = 12), which precludes definitive conclusions regarding equivalence. The high *p*-value primarily reflects the study’s limited power to detect clinically relevant differences. Larger, prospective comparative studies are needed. The IMPACT trial, evaluating the Tubridge device in posterior circulation aneurysms (*n* = 200), reported a 1-year complete occlusion rate of 79% with low rates of in-stent stenosis (3.6%) and symptomatic stroke (4.5%) (32). The device’s high metal coverage and optimized pore density are designed to profoundly alter flow dynamics. Notably, all four large aneurysms (≥ 13 mm) in our Tubridge cohort achieved complete occlusion, suggesting particular utility for complex morphologies. Similarly, the SEASE study of the Surpass Evolve device (*n* = 305) reported a 6-month occlusion rate of 73% with a major complication rate of 2.1% (33), corroborating the efficacy of modern FD designs. The single case of partial occlusion in a previously coiled aneurysm (OKM grade B) in our Pipeline group hints at potential challenges in such scenarios, possibly due to altered hemodynamics or a pro-inflammatory milieu impeding endothelialization.

The principal risks of FD treatment in the basilar territory—perforator ischemia, thromboembolism, and in-stent stenosis—were observed in our cohort as a 13.3% rate of transient ischemic symptoms. This aligns with the broad range of FD-related complication rates reported in the literature (0.8%–17.1%) (34, 35). Several factors likely contributed to our acceptable safety profile. First, the implementation of personalized antiplatelet therapy, guided by platelet function testing, was critical. In our cohort, 20% of patients identified as clopidogrel non-responders were switched to ticagrelor, an strategy supported by contemporary evidence (32, 36) for optimizing platelet inhibition and reducing thromboembolic risk. Second, advances in procedural technique, including the use of triaxial access systems and meticulous device sizing, contributed to safe deployment. Although 10% of patients developed jailed branch occlusions [similar to the 11%–19% reported with the FRED Jr. Device (36)], no acute branch occlusions or in-stent thromboses occurred. Emerging technologies like virtual deployment platforms (37) hold promise for further optimizing device placement and predicting hemodynamic outcomes.

Our study has several limitations that warrant consideration. First, the retrospective, single-center design and modest sample size (*n* = 30) limit the generalizability of our findings and the power for robust subgroup analyses, particularly for comparing devices. This is partly inherent to the study population, as the use of FDs for BAAs remains selective and is not yet a first-line treatment at many centers due to the complex anatomy and perceived risks. The mean angiographic follow-up of 12.9 months is insufficient to assess long-term durability, including risks of delayed in-stent stenosis or aneurysm recanalization. Second, the single-center design inherently introduces potential selection bias. Furthermore, when comparing our outcomes with historical data from other centers or earlier time periods, one must consider the potential influences of evolving patient selection criteria, advancements in device technology, refinements in antiplatelet regimens, and improved operator experience. These temporal and institutional differences may introduce bias when attempting direct comparisons. Furthermore, the exclusion of acutely ruptured and perforator-dominant aneurysms means our results are primarily applicable to carefully selected, unruptured BAAs. Future research should prioritize prospective, multicenter registries with long-term follow-up. Investigations into hybrid devices (e.g., combining branch-sparing stents with FDs), computational fluid dynamics for pre-procedural planning, and genotype-guided antiplatelet therapy represent promising avenues for further improving the safety and efficacy of FD treatment for these challenging lesions.

## 5 Conclusion

This single-center experience suggests that flow diverter devices represent a promising treatment modality for carefully selected patients with basilar artery aneurysms. The primary finding of this study is the achievement of technically successful deployment and favorable mid-term complete or near-complete occlusion rates (86.7%) with an acceptable safety profile in this complex anatomic location. The observed clinical outcomes underscore the importance of meticulous patient selection and preoperative planning. However, the small sample size, retrospective design, and lack of a control group preclude definitive conclusions regarding superiority over other treatments or generalizability. These preliminary findings warrant further validation through larger, prospective, and comparative studies to establish long-term efficacy and optimize patient selection criteria.

## Data Availability

The original contributions presented in the study are included in the article/supplementary material, further inquiries can be directed to the corresponding author.

## Glossary

ADP: adenosine diphosphate
AICA: anterior inferior cerebellar artery
BAA: basilar artery aneurysm
CI: confidence interval
CFD: computational fluid dynamics
CTA: computed tomography angiography
DSA: digital subtraction angiography
DynaCT: dynamic computed tomography
FD: flow diverter
FU: follow-up
mRS: modified Rankin Scale
MRA: magnetic resonance angiography
MRI: magnetic resonance imaging
OKM: O’Kelly–Marotta scale
OR: odds ratio
P2Y₁₂: purinergic receptor P2Y₁₂
PCA: posterior cerebral artery
PED: pipeline embolization device
SAC: stent-assisted coiling
SCA: superior cerebellar artery
SD: standard deviation
TFD: Tubridge flow diverter
TIA: transient ischemic attack
UIA: unruptured intracranial aneurysm
